# Inhibition of Nerve Growth Factor-Induced Neurite Outgrowth from PC12 Cells by Dexamethasone: Signaling Pathways through the Glucocorticoid Receptor and Phosphorylated Akt and ERK1/2

**DOI:** 10.1371/journal.pone.0093223

**Published:** 2014-03-25

**Authors:** Kazuki Terada, Yoshitsugu Kojima, Takayuki Watanabe, Nobuo Izumo, Koji Chiba, Yoshiharu Karube

**Affiliations:** 1 Laboratory of Drug Design and Drug Delivery, Faculty of Pharmaceutical Sciences, Fukuoka University, Fukuoka, Japan; 2 Laboratory of Clinical Pharmacology, Yokohama College of Pharmacy, Kanagawa, Japan; 3 Research and Development Department 2, Nippon Sigmax Co., Ltd., Tokyo, Japan; University of Pecs Medical School, Hungary

## Abstract

Glucocorticoids are important mediators of the stress response and are commonly employed as drugs for the suppression of immune rejection after organ transplantation. Previous investigations uncovered the possibility of mood depression in patients undergoing long-term treatment with synthetic glucocorticoids, including dexamethasone (DEX). Exogenous glucocorticoids and their synthetic derivatives can also adversely affect the development of the central nervous system. Although neurite extension from rat pheochromocytoma-derived PC12 cells and a variety of primary neurons is stimulated by nerve growth factor (NGF), and signaling pathways triggered by the binding of NGF to tyrosine kinase receptor type 1 (TrkA) function in both neurite outgrowth and neuronal survival, the effect of DEX on the activation of regulatory proteins and pathways downstream of TrkA has not been well characterized. To analyze the influence of DEX on NGF-induced neurite outgrowth and signaling, PC12 cells, a widely utilized model of neuronal differentiation, were pretreated with the glucocorticoid prior to NGF induction. NGF-induced neurite outgrowth was attenuated by pretreatment with DEX, even in the absence of DEX after the addition of NGF. Moreover, DEX suppressed the phosphorylation of Akt and extracellular-regulated kinase 1/2 (ERK1/2) in the neurite outgrowth signaling cascade initiated by NGF. Finally, the glucocorticoid receptor (GR) antagonist, RU38486, counteracted the inhibitory effect of DEX pretreatment, not only on the phosphorylation of Akt and ERK1/2, but also on neurite extension from PC12 cells. These results suggest that DEX binding to the GR impairs NGF-promoted neurite outgrowth by interfering with the activation/phosphorylation of Akt and ERK1/2. These novel findings are likely to be useful for elucidating the central nervous system depressive mechanism(s) of action of DEX and other glucocorticoids.

## Introduction

Glucocorticoids are critical mediators of the stress response in neurons and other cell types. Exposure of cells to stress triggers the glucocorticoid-mediated activation of corticotropin-releasing hormones, which in turn stimulate the synthesis of pituitary corticotropin as a part of the hypothalamo-pituitary adrenal axis [1]. Glucocorticoids are also involved in cell proliferation, neurotransmitter synthesis [2], neuronal survival, and neuronal differentiation [3], [4]. Clinically, synthetic glucocorticoids such as dexamethasone (DEX) are used for the suppression of immune rejection after organ transplantation and in the treatment of leukemia. However, the clinical use of synthetic glucocorticoids increases the risk of mood depression in patients receiving long-term therapy with these agents [5]. Furthermore, prenatal exposure to DEX can lead to the abnormal development of the central nervous system [6], [7].

Nerve growth factor (NGF) is a member of the neurotrophin family [8] that regulates cell proliferation and differentiation within specific neural tissues during physiological as well as pathological processes [9]. In particular, NGF is essential for cognitive function, and disrupted signaling through NGF is related to the development of Alzheimer’s disease and other neurodegenerative disorders [10]. The functions of NGF are mediated by two distinct receptor types, tyrosine kinase receptor type 1 (TrkA) and the p75 neurotrophin receptor (p75^NTR^) [11], [12]. TrkA is a high-affinity catalytic receptor for NGF, whereas p75^NTR^ is a low-affinity non-enzymatic NGF receptor. After NGF binds to TrkA, the receptor is subjected to cellular internalization, together with autophosphorylation of its tyrosine residues [13], [14]. Next, understream signaling, such as that mediated by phosphatidylinositol-3 kinase (PI3K) and activated Ras, takes place in the NGF-stimulated cells [15]–[17]. Thus, NGF binding to TrkA mediates axonal outgrowth and cellular survival in the affected neurons, whereas NGF binding to p75^NTR^ is hypothesized to antagonize TrkA-stimulated signal transduction [18].

The current study made use of the PC12 pheochromocytoma cell line to evaluate the capacity of DEX to interfere with neuronal signaling downstream of TrkA. PC12 cells provide a useful model for the investigation of neuronal differentiation, signaling, and other neurobiochemical/neurobiological events [19]–[22]. In the presence of NGF, PC12 cells differentiate into sympathetic neuron-like cells and are capable of substantial neurite outgrowth [23]. These properties allow for the exploration of possible cross-talk between glucocorticoid- and neurotrophin-activated signaling pathways that might affect the morphological differentiation of neurons. Indeed, PC12 cells have been successfully utilized to evaluate the effects of various natural and synthetic glucocorticoids on neurite outgrowth and related signaling cascades [24]–[27]. To our knowledge, however, no information is available regarding the DEX-induced modulation of protein activation/phosphorylation downstream of the TrkA receptor.

The present study now demonstrates that the interaction of DEX with the glucocorticoid receptor (GR) can inhibit the activation of Akt and extracellular-regulated kinase 1/2 (ERK1/2) following the binding of NGF to TrkA, effectively attenuating neurite outgrowth from PC12 cells. These findings are likely to shed light on the means by which DEX adversely affects the central nervous system.

## Materials and Methods

### Materials

DEX (dexamethasone sodium phosphate) was purchased from Wako Pure Chemical Industries, Ltd. (Osaka, Japan). NGF (murine NGF (mNGF) 2.5S derived from mouse submaxillary glands) was obtained from Alomone Labs, Ltd. (Jerusalem, Israel). The GR antagonist, RU38486, was purchased from Sigma-Aldrich (St. Louis, MO, USA).

### Cell Culture

PC12 cells were obtained from the Riken Cell Bank (Ibaraki, Japan). The cells were maintained in Dulbecco’s modified Eagle’s medium (DMEM) (Gibco-Life Technologies, Gaithersburg, MD, USA) supplemented with nutrient mixture F-12 (Gibco-Life Technologies), 10% (v/v) fetal bovine serum (FBS) (Gibco-Life Technologies), and 1% (v/v) penicillin/streptomycin (Nacalai Tesque, Kyoto, Japan). The cells were kept in an incubator at 37°C in an atmosphere of 5% CO_2_/95% air. Twenty-four hours prior to experimentation, the PC12 cells were seeded onto type I collagen-coated 60 mm tissue culture dishes (Iwaki, Tokyo, Japan) at a density of 1.5×10^5^ cells/dish in DMEM/F12 containing 10% FBS.

### Measurement of PC12 Cell Morphological Differentiation (Neurite Outgrowth)

PC12 cells were pretreated with DEX (1 μM) or vehicle (DMEM/F12 containing 10% FBS) for 24 h, washed with phosphate buffered saline (PBS), and stimulated with differentiation medium (DMEM/F12 containing 5% FBS plus 50 ng/mL NGF). Cells were incubated for an additional 24 h and photographed by using a digital camera (Digital Sight DS-L2 system; Nikon, Tokyo, Japan) under a phase-contrast microscope (ECLIPSE TS100; Nikon).

Images of five randomly selected fields per dish were obtained that included a mean number of 10–20 PC12 cells per field. The total length of the neurites derived from all 10–20 cells in each of the five fields was automatically measured by using Neurocyte Image Analyzer Software (Kurabo, Osaka, Japan). The average neurite length per field was obtained by dividing the total neurite length by the number of cells per field. Finally, the results from all of the fields were summed and divided by the total number of fields (n = 5) to yield the average neurite length per condition.

### Western Blotting Analysis

PC12 cells were pretreated with DEX (1 μM) for 24 h, washed with PBS, and stimulated with NGF (50 ng/mL) in DMEM/F12 containing 5% FBS. At selected time points (0, 5, 10, 15, 30, and 60 min) following the addition of NGF, the cells were harvested in ice-cold Tris-buffered saline (TBS) (Bio-Rad, Hercules, CA, USA) and lysed with PathScan Sandwich ELISA Lysis Buffer (Cell Signaling Technology, Beverly, MA, USA) on ice for 5 min. Cell debris was removed by centrifugation, and protein concentrations in the lysates were determined by using the Bicinchoninic Acid (BCA) Assay Kit (Pierce, Rockford, IL, USA). Protein samples containing equal amounts of protein were separated by electrophoresis in sodium dodecyl sulfate (SDS)-polyacrylamide gels, followed by electrophoretic transfer onto polyvinylidene fluoride (PVDF) membranes. The PVDF membranes were blocked with 5% bovine serum albumin (Wako Pure Chemical Industries, Ltd.) in TBS containing 0.1% Tween-20 at room temperature for 1 h.

Next, immunoblotting was performed by using primary anti-phospho-Akt (p-Akt) antibody (Ser473; Cell Signaling Technology), primary anti-Akt antibody (Cell Signaling Technology), primary anti-phospho-ERK1 (T202/Y204)/ERK2 (T185/Y187) (p-ERK1/2) antibody (R&D Systems, Minneapolis, MN, USA), and primary anti-ERK1/2 antibody (R&D Systems). Immunoreactive bands were detected by reaction with a horseradish peroxidase-conjugated secondary antibody (Amersham Pharmacia Biotech, Piscataway, NJ, USA) and visualized by using enhanced chemiluminescence Western blotting detection reagents (Amersham Pharmacia Biotech) and RX-U Fuji X-ray film (Fuji Film, Tokyo, Japan). ImageJ software (freely available from the National Institutes of Health, Bethesda, MD, USA) was employed for data analysis.

### Pharmacological Inhibition of the GR in PC12 Cells

To evaluate the influence of GR blockade on the DEX-mediated regulation of neurite outgrowth, PC12 cells were pretreated with the glucocorticoid (1 μM) and RU38486 (2 μM), a pharmacological inhibitor of the GR, at 24 h prior to the addition of NGF (50 ng/mL). The cells were incubated with NGF for another 24 h, and neurite outgrowth was then analyzed as described above. Alternatively, the cells were harvested at 10 min after the addition of NGF for Western blotting analysis with primary anti-p-Akt, anti-Akt, anti-p-ERK1/2, and anti-ERK1/2 antibodies.

### Statistical Analysis

Quantitative data are given as the means ± the standard deviation (SD). Statistical analysis of the quantitative data was performed by applying an analysis of variance (ANOVA) test followed by Tukey’s post-hoc test. In all cases, *P<*0.05 was considered statistically significant.

## Results

### DEX Attenuates NGF-induced Neurite Outgrowth from PC12 Cells

**Figure 1:**
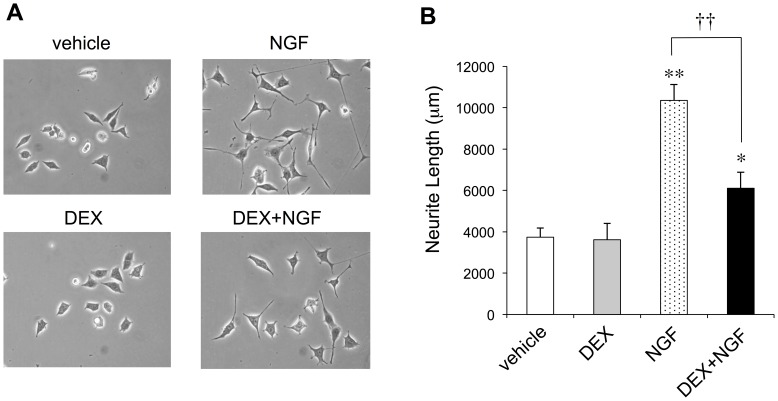
DEX pretreatment attenuates NGF-induced promotion of neurite outgrowth from PC12 cells. PC12 cells were pretreated for 24(1 μM). They were then stimulated with NGF for another 24 h. (**A**) Images of the cultures are shown at 400× magnification. (**B**) Neurite lengths were analyzed as described in the Materials and Methods section. The values represent the means ± the SD for all PC12 cells included in five randomly chosen fields per condition (*P<0.05, DEX+NGF versus vehicle; **P<0.01, NGF versus vehicle; ^††^ *P*<0.01, DEX+NGF versus NGF).

PC12 cells extended few neurites in the absence of NGF (Fig. **1A**). As expected, total neurite outgrowth was significantly increased by treatment with the neurotrophin (Fig. **1A, B**). However, the NGF-induced increase in neuronal process extension was significantly attenuated by DEX pretreatment (DEX+NGF). In the absence of NGF, no differences were observed between the vehicle (DMEM/F12/10% FBS) group and the DEX group (Fig. **1A, B**). Therefore, DEX effectively inhibited NGF-induced neurite outgrowth from PC12 cells.

### DEX Blocks NGF-induced Activation/phosphorylation of Akt and ERK1/2

**Figure 2:**
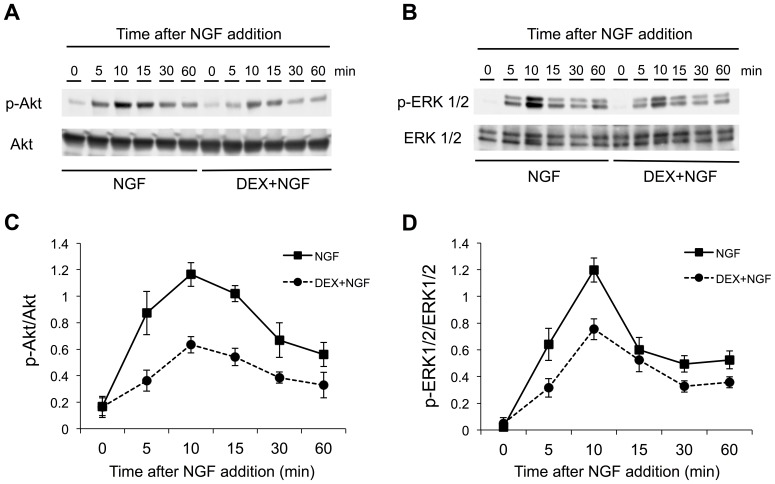
DEX pretreatment inhibits NGF-induced phosphorylation of Akt and ERK1/2. PC12 cells were pretreated for 24(1 μM). They were then stimulated with NGF for varying periods of time (0, 5, 10 15, 30, or 60 min). (**A, B**) Whole cell extracts were prepared and subjected to SDS-polyacrylamide gel electrophoresis, transferred to PVDF membranes, and immunoblotted with primary anti-p-Akt antibody and anti-Akt antibody (A), as well as with primary anti-p-ERK1/2 antibody and anti-ERK1/2 antibody (B), followed by a horseradish peroxidase-conjugated secondary antibody**.** (**C, D**) The intensity of the p-Akt polypeptide band was normalized to that of the total Akt band (p-Akt/Akt) (C), and the intensity of the p-ERK1/2 polypeptide band was normalized to that of the total ERK1/2 band (p-ERK1/2/ERK1/2) (D). Results are presented as the means ± the SD for three independent experiments.

The time course of signaling activation associated with NGF-induced neurite outgrowth was next assessed in PC12 cells with or without DEX pretreatment. This was done by analyzing the phosphorylation status of Akt and ERK1/2 on Western blots. After the addition of NGF to the cells without DEX pretreatment, the protein levels of phosphorylated Akt (p-Akt) gradually increased and reached their peak 10 min later (Fig. **2A, C**). The p-Akt levels then declined by 52% at 60 min after NGF treatment (Fig. **2C**). With DEX pretreatment, p-Akt levels also peaked in PC12 cells at 10 min after NGF treatment and declined thereafter (Fig. **2A, C**), but the p-Akt content was about 41–59% lower in the presence versus the absence of DEX at each time point examined (Fig. **2C**). This was not due to lower amounts of total protein in the DEX-pretreated versus non-pretreated samples because equal amounts of protein were loaded into each lane of the SDS-PAGE gel, as assessed by the BCA assay.

Similarly, the amount of phosphorylated ERK1/2 (p-ERK1/2) was highest at 10 min after the addition of NGF without DEX pretreatment (Fig. **2B, D**). However, DEX pretreatment reduced ERK1/2 phosphorylation by ∼37% at the same time point (Fig. **2D**). These results demonstrate that exposure of PC12 cells to DEX prior to neurotrophin treatment downregulated the NGF-induced stimulation of Akt and ERK1/2.

### RU38486 Prevents DEX-mediated Inhibition of NGF-induced Neurite Outgrowth and Akt/ERK1/2 Phosphorylation

**Figure 3:**
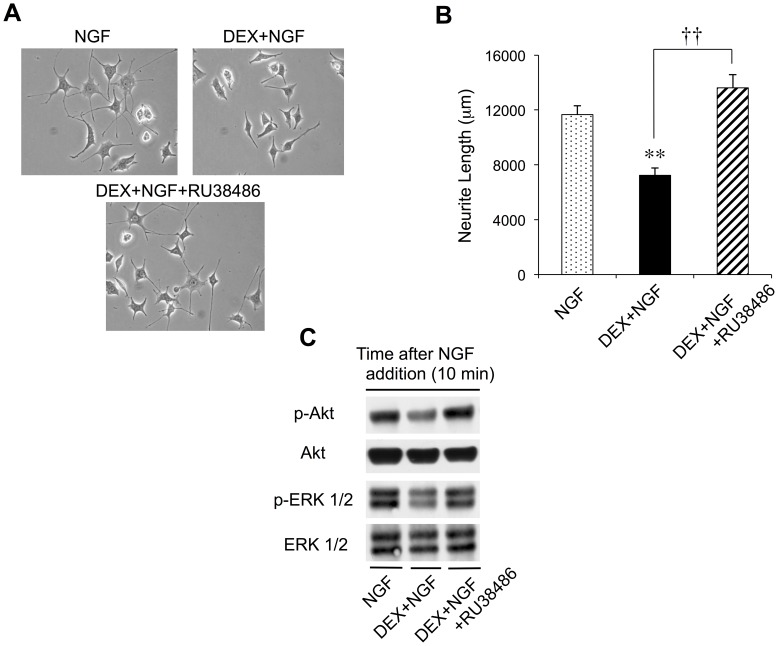
RU38486 reverses DEX-mediated attenuation of NGF-induced neurite outgrowth and Akt/ERK1/2 phosphorylation. PC12 cells were pretreated for 24(**A**) Images of the cells are shown at 400× magnification. (**B**) Neurite lengths were analyzed as described in the Materials and Methods section. The values represent the means ± the SD for all PC12 cells included in five randomly chosen fields per condition (**P<0.01, DEX+NGF versus NGF; ^††^ *P*<0.01, DEX+NGF versus DEX+NGF+RU36486). (**C**) Whole cell extracts were subjected to SDS-polyacrylamide gel electrophoresis, transferred to PVDF membranes, and immunoblotted with primary anti-p-Akt, anti-Akt, anti-p-ERK1/2, and anti-ERK1/2 antibodies, followed by a horseradish peroxidase-conjugated secondary antibody.

We next investigated the ability of RU38486, a GR antagonist, to interfere with the DEX-mediated attenuation of NGF-induced neurite outgrowth and Akt/ERK1/2 signaling activation. PC12 cells were pretreated with vehicle, DEX, or DEX together with RU38486 for 24 h. NGF was then added and incubated with the cultures for another 24 h, at which time total neurite outgrowth was assessed. As seen above (Fig. **1A, B**), NGF facilitated neurite extension from PC12 cells, while DEX reversed the actions of NGF (Fig. **3A, B**). The inhibition of NGF-induced neurite outgrowth by DEX pretreatment was significantly counteracted by simultaneous pretreatment with RU38486 (Fig. **3A, B**). Furthermore, RU38486 completely abolished the capacity of DEX to interfere with NGF-induced Akt and ERK1/2 phosphorylation. Figure **3C** shows that the concomitant pretreatment of PC12 cells with RU38486 plus DEX restored the NGF-induced phosphorylation of both signaling molecules at 10 min after the addition of the neurotrophin, when the peak levels of p-Akt and p-ERK1/2 were initially observed (Fig. **2**). These findings suggest that the mitigation of neurite outgrowth by DEX occurred through DEX interaction with the GR, followed by the inhibition of Akt and ERK1/2 phosphorylation, and/or the activation of signaling proteins upstream of Akt and ERK1/2.

## Discussion

The present study utilized PC12 cells as a neuronal model to demonstrate that NGF-induced neurite outgrowth, a hallmark of neuronal morphological differentiation, was blocked by pretreatment of the cells with a synthetic glucocorticoid, DEX (Fig. **1**). DEX-mediated interference with neurite outgrowth was accompanied by specific neurobiochemical changes, as evidenced by the downregulation of phosphorylated Akt and ERK1/2 levels in the NGF-treated cultures (Fig. **2**). Moreover, the current investigation utilized a specific GR antagonist, RU38486, to show that the actions of DEX to modulate neurite outgrowth and neurite outgrowth-associated signaling pathways were actually facilitated *via* the GR (Fig. **3**). To our knowledge, this is the first report to demonstrate that the interaction of DEX with the GR, the phosphorylation/dephosphorylation of Akt and ERK1/2, and NGF-induced neurite outgrowth are all interrelated.

**Figure 4:**
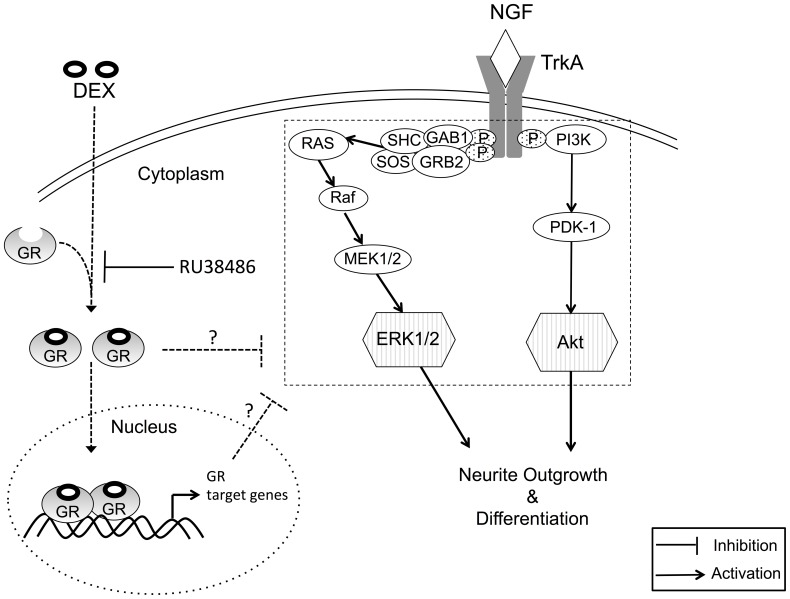
Proposed scheme of the signaling pathways involved in DEX-mediated inhibition of NGF-induced neurite outgrowth and Akt/ERK1/2 phosphorylation/activation. DEX associates with the GR in the cytoplasm and inhibits the phosphorylation of signaling molecules (e.g., Ras/ERK1/2 and PI3K/Akt) downstream of the TrkA receptor. DEX-mediated inhibition of NGF-induced neurite outgrowth is proposed to occur by preventing the phosphorylation of Akt and/or ERK1/2 and/or upstream proteins (e.g., Ras and PI3K).

The mechanism underlying NGF-induced neurite outgrowth from PC12 cells has been investigated in various studies [9], [28], [29]. In the cascade of signaling events contributing to the morphological differentiation of PC12 cells, Ras and PI3K signaling events are absolutely pivotal to the control of NGF-induced neurite extension [9], [30], [31]. Akt and ERK1/2 are then activated downstream of Ras and PI3K, respectively (Fig. **4**) [32], [33]. A significant increase in total neurite outgrowth from PC12 cells by the addition of NGF was also observed in the present study (Figs. **1** and **3**), along with phosphorylation/activation of Akt and ERK1/2 (Figs. **2** and **3**). As noted above, the NGF-induced enhancement of neurite outgrowth and phosphorylation of Akt and ERK1/2 were both attenuated by DEX pretreatment of PC12 cells (Figs. **1**, **2**, and **3**). Pollock et al., however, provided controversial results in which DEX did not prevent neurite outgrowth from PC12 cells in the continued presence of NGF [24]. On the other hand, Lecht et al. demonstrated that NGF-induced neurite outgrowth was inhibited in the presence of DEX [27], consistent with our observations.

Under the present experimental conditions (i.e., DEX pretreatment for 24 h followed by the washing out of DEX and the addition of NGF), DEX was undeniably inhibitory to neurite outgrowth. Because the inhibition of NGF-induced neurite outgrowth and Akt/ERK1/2 phosphorylation were both observed with DEX pretreatment, even after the removal of DEX, we propose that the DEX does not directly interact with the NGF TrkA receptor, but instead indirectly impedes the signaling activation of Akt and ERK1/2 by NGF. Moreover, the DEX-mediated inhibition of both of these processes was counteracted by the addition of RU38486 (Fig. **3**), demonstrating that DEX binding to the GR regulates the signaling activation of Akt and ERK1/2 and/or upstream proteins, such as PI3K and Ras. Importantly, Lecht et al. showed that DEX precipitated a 50% decrease in the selective binding of NGF to PC12 cells [27]. The capacity of DEX to affect signaling upstream of Akt and ERK1/2, but downstream of NGF binding to the TrkA receptor, could explain the results of both Lecht et al. and Pollock et al., as well as the indirect inhibition of TrkA-mediated signaling by the glucocorticoid.

Lecht and colleagues also reported that DEX can reduce the mRNA and protein expression levels of the low-affinity NGF receptor, p75^NTR^, and that RU38486 reversed these actions [27]. The authors demonstrated that the downregulation of p75^NTR^ was associated with an increase in TrkA autophosphorylation/activation in PC12-6.24-I cells, a PC12 cell clone that constitutively overexpresses the TrkA receptor. Given that Akt and ERK1/2 act downstream of autophosphorylated TrkA, these results are seemingly inconsistent with our findings of the DEX-mediated inhibition of NGF-induced activation of Akt and ERK1/2. The discrepancy might be explained by the difference in cell types and/or divergent experimental conditions between the present study and that of Lecht et al. Moreover, Lecht et al. addressed the relationship between DEX-mediated inhibition of NGF-induced neurite outgrowth and the elevated levels of autophosphorylated TrkA, and proposed that an as yet unknown regulatory process might contribute to the uncoupling of TrkA activation and TrkA-mediated neurite outgrowth. However, further research in this area is required.

Another investigation reported that wortmannin, an inhibitor of PI3K, obstructed the NGF-induced activation of Akt [34]. Furthermore, lovastatin, an inhibitor of Ras, blocked the NGF-induced activation of ERK1/2 and neurite outgrowth [35], [36]. These results suggest that the signaling activation of PI3K, Akt, and ERK1/2 is linked to the promotion of neurite outgrowth by NGF from PC12 cells (Fig. **4**). However, we found that the simultaneous addition of DEX and NGF to PC12 cells failed to inhibit the signaling activation of Akt and ERK1/2 (data not shown). This observation could be explained by the time lag between the signaling from the GR to the protein phosphorylation of Akt and ERK1/2 and/or upstream signaling molecules.

In conclusion, this study demonstrated that DEX pretreatment of PC12 cells inhibited NGF-induced neurite outgrowth. DEX apparently exerted its actions by binding to the GR and by subsequently preventing the NGF-stimulated phosphorylation/activation of Akt and ERK1/2, perhaps via actions on upstream effector proteins such as PI3K and Ras. Because DEX is well known to cause mood depression with long-term use [37], the present findings may prove useful in elucidating the central nervous system depression mechanism of action of the synthetic glucocorticoid.

## References

[1] Wang Q, Van Heerikhuize J, Aronica E, Kawata M, Seress L, et al (2013) Glucocorticoid receptor protein expression in human hippocampus; stability with age. Neurobiol Aging 34: 1662–1673. 23290588 10.1016/j.neurobiolaging.2012.11.019

[2] McEwen BS, De Kloet ER, Rostene W (1986) Adrenal steroid receptors and actions in the nervous system. Physiol Rev 66: 1121–1188. 3532143 10.1152/physrev.1986.66.4.1121

[3] Gould E, Tanapat P (1999) Stress and hippocampal neurogenesis. Biol Psychiatry 46: 1472–1479. 10599477 10.1016/s0006-3223(99)00247-4

[4] Glick RD, Medary I, Aronson DC, Scotto KW, Swendeman SL, et al (2000) The effects of serum depletion and dexamethasone on growth and differentiation of human neuroblastoma cell lines. J Pediatr Surg 35: 465–472. 10726691 10.1016/s0022-3468(00)90216-1

[5] Celano CM, Freudenreich O, Fernandez-Robles C, Stern TA, Caro MA, et al (2011) Depressogenic effects of medications: a review. Dialogues Clin Neurosci 13: 109–125. 21485751 10.31887/DCNS.2011.13.1/ccelano PMC3181967

[6] Gilstrap LC, Christensen R, Clewell WH, D’Alton ME, Davidson EC, et al (1995) Effect of corticosteroids for fetal maturation on perinatal outcomes. NIH Consensus Development Panel on the Effect of Corticosteroids for Fetal Maturation on Perinatal Outcomes. Jama 273: 413–418. 7823388 10.1001/jama.1995.03520290065031

[7] Davis EP, Sandman CA, Buss C, Wing DA, Head K (2013) Fetal glucocorticoid exposure is associated with preadolescent brain development. Biol Psychiatry 74: 647–655. 23611262 10.1016/j.biopsych.2013.03.009 PMC3985475

[8] Schaper C, Glaser S, Groneberg DA, Kunkel G, Ewert R, et al (2009) Nerve growth factor synthesis in human vascular smooth muscle cells and its regulation by dexamethasone. Regul Pept 157: 3–7. 19596029 10.1016/j.regpep.2009.07.004

[9] Sofroniew MV, Howe CL, Mobley WC (2001) Nerve growth factor signaling, neuroprotection, and neural repair. Annu Rev Neurosci 24: 1217–1281. 11520933 10.1146/annurev.neuro.24.1.1217

[10] Wigerius M, Asghar N, Melik W, Johansson M (2013) Scribble controls NGF-mediated neurite outgrowth in PC12 cells. Eur J Cell Biol 92: 213–221. 23973368 10.1016/j.ejcb.2013.07.002

[11] Chao MV (1992) Neurotrophin receptors: a window into neuronal differentiation. Neuron 9: 583–593. 1327010 10.1016/0896-6273(92)90023-7

[12] Barbacid M (1994) The Trk family of neurotrophin receptors. J Neurobiol 25: 1386–1403. 7852993 10.1002/neu.480251107

[13] Zhang Y, Moheban DB, Conway BR, Bhattacharyya A, Segal RA (2000) Cell surface Trk receptors mediate NGF-induced survival while internalized receptors regulate NGF-induced differentiation. J Neurosci 20: 5671–5678. 10908605 10.1523/JNEUROSCI.20-15-05671.2000 PMC6772538

[14] Geetha T, Jiang J, Wooten MW (2005) Lysine 63 polyubiquitination of the nerve growth factor receptor TrkA directs internalization and signaling. Mol Cell 20: 301–312. 16246731 10.1016/j.molcel.2005.09.014

[15] Kaplan DR, Stephens RM (1994) Neurotrophin signal transduction by the Trk receptor. J Neurobiol 25: 1404–1417. 7852994 10.1002/neu.480251108

[16] Greene LA, Kaplan DR (1995) Early events in neurotrophin signalling via Trk and p75 receptors. Curr Opin Neurobiol 5: 579–587. 8580709 10.1016/0959-4388(95)80062-x

[17] Yao R, Cooper GM (1995) Requirement for phosphatidylinositol-3 kinase in the prevention of apoptosis by nerve growth factor. Science 267: 2003–2006. 7701324 10.1126/science.7701324

[18] Huang EJ, Reichardt LF (2003) Trk receptors: roles in neuronal signal transduction. Annu Rev Biochem 72: 609–642. 12676795 10.1146/annurev.biochem.72.121801.161629

[19] Guroff G (1985) PC12 Cells as a Model of Neuronal Differentiation. Cell Culture in the Neurosciences 1: 245–272.

[20] Rossi D, Pedrali A, Gaggeri R, Marra A, Pignataro L, et al (2013) Chemical, pharmacological, and in vitro metabolic stability studies on enantiomerically pure RC-33 compounds: promising neuroprotective agents acting as sigma(1) receptor agonists. ChemMedChem 8: 1514–1527. 23832823 10.1002/cmdc.201300218

[21] Yu CW, Chang PT, Hsin LW, Chern JW (2013) Quinazolin-4-one derivatives as selective histone deacetylase-6 inhibitors for the treatment of Alzheimer's disease. J Med Chem 56: 6775–6791. 23905680 10.1021/jm400564j

[22] Nishina A, Kimura H, Tsukagoshi H, Kozawa K, Koketsu M, et al (2013) Neurite outgrowth of PC12 cells by 4′-O-beta-D-glucopyranosyl-3′,4- dimethoxychalcone from Brassica rapa L. ‘hidabeni’ was enhanced by pretreatment with p38MAPK inhibitor. Neurochem Res 38: 2397–2407. 24057400 10.1007/s11064-013-1152-7

[23] Das KP, Freudenrich TM, Mundy WR (2004) Assessment of PC12 cell differentiation and neurite growth: a comparison of morphological and neurochemical measures. Neurotoxicol Teratol 26: 397–406. 15113601 10.1016/j.ntt.2004.02.006

[24] Pollock JD, Krempin M, Rudy B (1990) Differential effects of NGF, FGF, EGF, cAMP, and dexamethasone on neurite outgrowth and sodium channel expression in PC12 cells. J Neurosci 10: 2626–2637. 2167354 10.1523/JNEUROSCI.10-08-02626.1990 PMC6570261

[25] Hughes AL, Gollapudi L, Sladek TL, Neet KE (2000) Mediation of nerve growth factor-driven cell cycle arrest in PC12 cells by p53. Simultaneous differentiation and proliferation subsequent to p53 functional inactivation. J Biol Chem 275: 37829–37837. 10978315 10.1074/jbc.M003146200

[26] Perrone-Bizzozero NI, Cansino VV, Kohn DT (1993) Posttranscriptional regulation of GAP-43 gene expression in PC12 cells through protein kinase C-dependent stabilization of the mRNA. J Cell Biol 120: 1263–1270. 8436593 10.1083/jcb.120.5.1263 PMC2119722

[27] Lecht S, Arien-Zakay H, Tabakman R, Jiang H, Fink DW, et al (2007) Dexamethasone-Induced Down-Regulation of Nerve Growth Factor Receptor p75NTR is Mediated by Glucocorticoid Type II Receptor in PC12 Cell Model. Open Pharmacol J 1: 19–26.

[28] Greene LA, Burstein DE, Black MM (1982) The role of transcription-dependent priming in nerve growth factor promoted neurite outgrowth. Dev Biol 91: 305–316. 7095268 10.1016/0012-1606(82)90037-9

[29] Rossner S, Ueberham U, Schliebs R, Perez-Polo JR, Bigl V (1998) p75 and TrkA receptor signaling independently regulate amyloid precursor protein mRNA expression, isoform composition, and protein secretion in PC12 cells. J Neurochem 71: 757–766. 9681467 10.1046/j.1471-4159.1998.71020757.x

[30] Kimura K, Hattori S, Kabuyama Y, Shizawa Y, Takayanagi J, et al (1994) Neurite outgrowth of PC12 cells is suppressed by wortmannin, a specific inhibitor of phosphatidylinositol 3-kinase. J Biol Chem 269: 18961–18967. 8034653

[31] Marshall CJ (1995) Specificity of receptor tyrosine kinase signaling: transient versus sustained extracellular signal-regulated kinase activation. Cell 80: 179–185. 7834738 10.1016/0092-8674(95)90401-8

[32] Chambard JC, Lefloch R, Pouyssegur J, Lenormand P (2007) ERK implication in cell cycle regulation. Biochim Biophys Acta 1773: 1299–1310. 17188374 10.1016/j.bbamcr.2006.11.010

[33] Manning BD, Cantley LC (2007) AKT/PKB signaling: navigating downstream. Cell 129: 1261–1274. 17604717 10.1016/j.cell.2007.06.009 PMC2756685

[34] Kim Y, Seger R, Suresh Babu CV, Hwang SY, Yoo YS (2004) A positive role of the PI3-K/Akt signaling pathway in PC12 cell differentiation. Mol Cells 18: 353–359. 15650333

[35] Cerezo-Guisado MI, Garcia-Roman N, Garcia-Marin LJ, Alvarez-Barrientos A, Bragado MJ, et al (2007) Lovastatin inhibits the extracellular-signal-regulated kinase pathway in immortalized rat brain neuroblasts. Biochem J 401: 175–183. 16952276 10.1042/BJ20060731 PMC1698684

[36] Price RD, Yamaji T, Matsuoka N (2003) FK506 potentiates NGF-induced neurite outgrowth via the Ras/Raf/MAP kinase pathway. Br J Pharmacol 140: 825–829. 14559856 10.1038/sj.bjp.0705522 PMC1574111

[37] Hall RC, Popkin MK, Kirkpatrick B (1978) Tricyclic exacerbation of steroid psychosis. J Nerv Ment Dis 166: 738–742. 702131

